# Photon-counting detector CT angiography to evaluate carotid and subclavian artery stents and compared to ultrasound and angiography – an in-vivo study with spectral reconstructions

**DOI:** 10.1177/15910199251374274

**Published:** 2025-09-08

**Authors:** Frederic De Beukelaer, Laura L Wuyts, Steven van Hedent, Omid Nikoubashman, Iliana Kantzeli, Martin Wiesmann, Arno Reich, João Pinho, Hani Ridwan, Charlotte S Weyland

**Affiliations:** 1 Department of Neuroradiology, 39058 University Hospital RWTH Aachen, Aachen, Germany; 2 Department of Radiology, 82218 AZ Sint-Lucas, Gent, Belgium; 3 Department of Neurology, 364965 University Hospital RWTH Aachen, Aachen, Germany

**Keywords:** CT-angiography, stent, DSA, duplex sonography, photon counting CT

## Abstract

**Purpose:**

To evaluate the potential of Photon-Counting Detector CT Angiography (PCD-CTA) for the assessment of carotid and subclavian artery stents compared to digital subtraction angiography (DSA) and Duplex ultrasound (DUS).

**Methods:**

This study is a single-center, retrospective analysis of consecutive patients treated with a stent for high grade stenosis of the extra-cranial carotid and the subclavian artery between April 2023 and May 2024. Polyenergetic images (PE), iodine and virtual monoenergetic images were performed at different keV levels (40 and 80) and with two body vascular reconstruction kernels (Bv56 and 72) with and without iterative metal artifact reduction. Three independent readers assessed image quality using a 5-point Likert scale and region of interest analysis. A blinded, independent reading was performed to determine in-stent vessel stenosis.

**Results:**

A total of 19 patients (64.3 ± 10.3 (mean patient age in years ± SD); 9 women) with carotid or subclavian artery stents and available DSA, DUS and PCD-CTA were analyzed. Virtual monoenergetic images (VMI) reconstructed with Bv56 at 40 keV, PE and IOD reconstructed with Bv56 scored higher and achieved higher SNRs and CNRs in the in-stent vessel lumen compared with Bv72 reconstructions (p < 0.001). In 2/19 cases with elevated flow velocities (>250 cm/s) in the in-stent vessel lumen, the subsequently performed PCD-CTA and DSA could rule out a high-grade stenosis.

**Conclusion:**

PCD-CTA with spectral reconstructions allows a reliable non-invasive assessment of the in-stent vessel lumen in patients after carotid artery or subclavian artery stenting.

## Introduction

Carotid Artery stenting (CAS) as well as subclavian artery stenting is an established therapy option to treat relevant subclavian artery and carotid artery stenosis.^1^ Restenosis is attributable to neointimal hyperplasia during the early postoperative period and has been shown to occur in 10.2% to 30.7% of cases.^2 [Bibr bibr3-15910199251374274] -4^

While non-invasive assessment of subclavian and brachiocephalic trunk stents requires CT-Angiography, extra-cranial carotid stents are evaluated using duplex ultrasound (DUS) with a high negative predictive value for carotid stenosis. Although DUS criteria for high-grade in-stent-restenosis (>70%) are heterogeneous, the largest population sample so far, using DSA as reference standard, recommends using primarily the peak systolic velocity of >300 cm/s.^5^ However, a recent publication has mentioned a peak systolic velocity of >250 m/s as the threshold for a flow-relevant in-stent-restenosis.^6^

DUS is limited by stent ends near the skull base and in heavily scarred tissue. Furthermore, US-velocity measurement is sensitive to inter-observer heterogeneity and can be hampered by stent artifacts.^1^

The common Energy-integrating Detector CT (EID-CT) is affected by blooming artifacts causing artificial lumen narrowing and leading to stenosis overestimation.^7^

To overcome these limitations, Dual-Energy CT and recently Photon-Counting-CTA (PCD-CTA) allow for improved vessel lumen visualization by using spectral reconstructions as virtual monoenergetic imaging (VMI) and iodine (IOD) reconstructions to evaluate coronary stents and intracranial stents.^8,9^

We studied all possible Photon Counting methods of image post-processing, integrating spectral reconstructions, useful kernels and keV-levels to identify the optimal reconstruction to evaluate the in-stent vessel lumen in carotid and subclavian stents as well as to investigate the diagnostic accuracy of PCD-CTA for the evaluation of in-stent vessel segments compared to digital subtraction angiography (DSA) and DUS.

## Methods

This study is a single-center, retrospective analysis of consecutive patients treated with a stent for high grade stenosis of the extra-cranial carotid arteries and the subclavian artery between April 2023 and May 2024. Imaging was performed additional to ultrasound evaluation in the clinical routine in case of needed second imaging modality.

The local ethics committee of the medical faculty of RWTH Aachen, Aachen, Germany (local registration number: EK 24-270) approved the study. Patient consent was waived due to the retrospective nature of the study design. The study is reported in adherence to the STROBE criteria.^10^

### Percutaneous transluminal angioplasty with stent placement

The indication for stent placement in the study cohort was made based on symptoms and patient constitution as well as on national and international guidelines.^11^ In the study period, all patients were treated with a trans-femoral access for carotid artery stenting and with a transradial or transbrachial access for subclavian artery stenting. Balloon-angioplasty was performed when necessary. The stent used for carotid arteries were Carotid WallStents^®^ (Boston Scientific, Marlborough, Massachusetts, USA) and CGuard^®^ (Inspire MD, Boston, MA, USA) and the stent used for subclavian arteries was Omnilink Elite^®^ Vascular Balloon-Expandable Stents (Abbott Laboratories, Abbott Park, IL, USA) sized according to the vessel diameters.

### Photon-counting CT imaging

All patients were scanned with a dual-source photon-counting CT scanner (NAEOTOM Alpha, Siemens Healthineers^®^, Erlangen, Germany, software version syngo CT VB10) operated in ultra-high-resolution mode. The imaging protocol consisted of an ECG-gated contrast-enhanced PCD-CTA, centered on the implanted device and triggered by bolus tracking. Specific scanning parameters are provided in the electronic Supplementary Materials.

**Figure 1.**
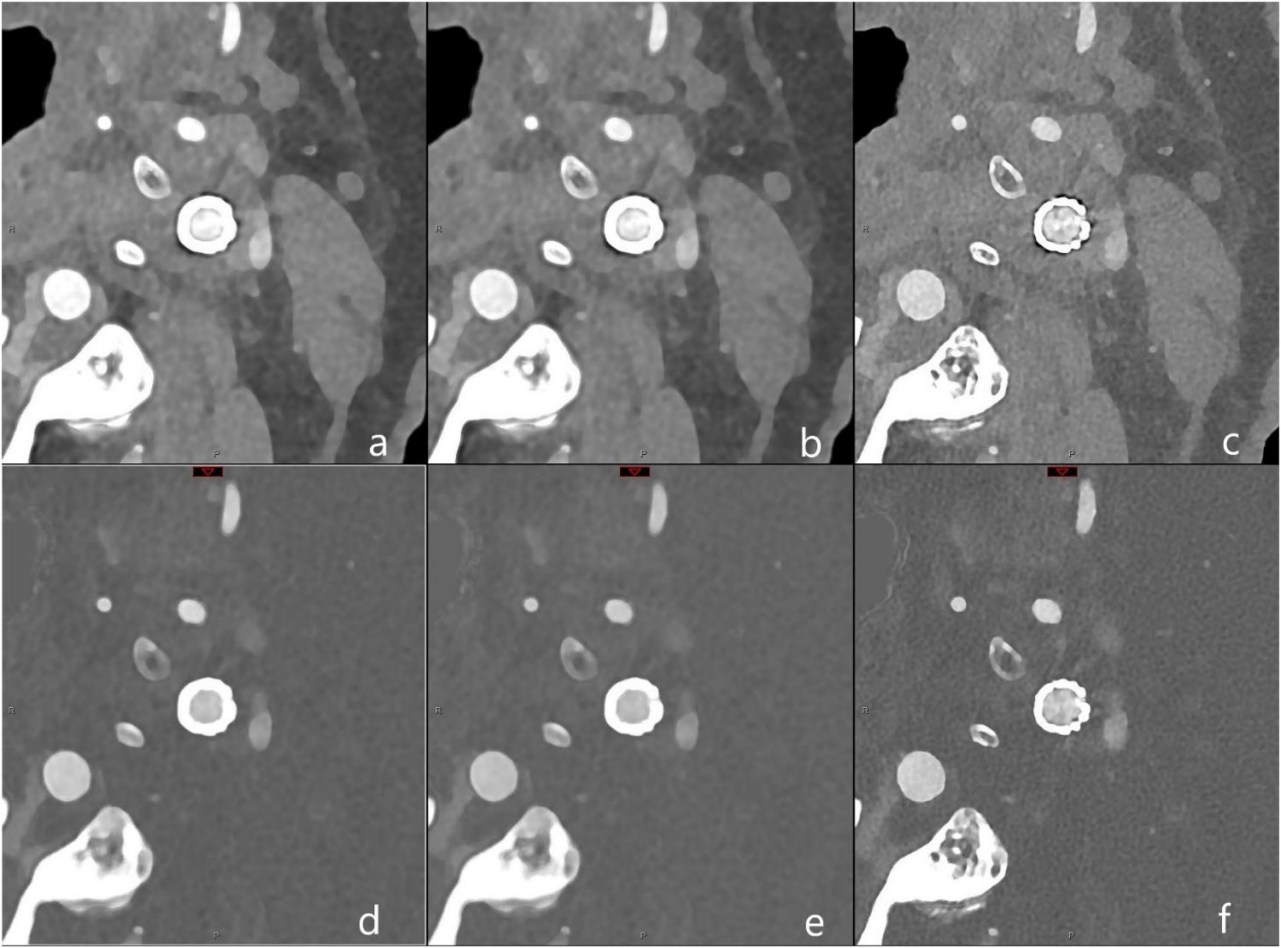
Illustration of reconstruction protocol for polyenergetic (PE) and iodine (IOD) reconstructions with two different kernels. In the first row PE and in the second row IOD are listed and in the three columns, from left to right Bv56 kernel without iMAR, Bv56 with iMAR and Bv72. In detail: a = Bv56, PE; b = Bv56 with iMAR, PE; c = Bv72, PE; d = Bv56, IOD; e = Bv56 with iMAR, IOD; f = Bv72, IOD. Abbreviations: Bv: Body vascular kernel, iMAR: iterative metal artifact reduction.

Polyenergetic (PE) and iodine reconstructions (IOD) were performed with two different body vascular (Bv) kernels (Bv56 and Bv72), virtual monoenergetic imaging (VMI) reconstructions were performed using the common Bv kernel and with two different kiloelectronvolts (keV) levels (40 and 80) as visualized in Figure 1. Reconstructions with Bv56 were additionally processed using iterative metal artifact reduction (iMAR) for dental implants.

Detailed information on the PCD-CTA image acquisition and post-processing, including spectral reconstruction specifics, can be found in the Supplementary Data.

Baseline demographic and clinical characteristics of the included patients were collected from the clinical records.

### Subjective image quality assessment

Three radiologists with 11 years (LW, FDB) and 12 years (SvH) of experience independently evaluated all images using a Syngo.via workstation (version VB10A, Siemens Healthineers) while remaining blinded to clinical data, as well as DSA and DUS results. Image quality was rated using a 5-point Likert scale (1 = “non-diagnostic,” 5 = “excellent”) based on lumen visibility in the vessel proximal of the stent and in the narrowest part of the in-stent vessel.

### Objective image quality assessments

Objective metrics, including contrast-to-noise ratios (CNR) and signal-to-noise ratios (SNR) are detailed in the online Supplementary Materials (Table S1). Three radiologists independently placed four regions of interest (ROI) in each reconstruction: one in the parent vessel within 1 cm proximal to the stent entry, one in the in-stent vessel lumen with the highest degree of stenosis, one in the air, and one in the sternocleidomastoid muscle. The mean Hounsfield units (HU) were recorded from these ROIs.

SNR and CNR were calculated as follows:

$$\mathrm{SNR}=\frac{{\mathrm{signal}}_{\mathrm{artery}}}{\mathrm{standard}\;\mathrm{deviatio}n_{\mathrm{artery}}}$$

$$\mathrm{CNR}=\frac{{\mathrm{signal}}_{\mathrm{artery}}\text{–}\mathrm{signa}l_{\mathrm{muscle}}}{\mathrm{standard}\;\mathrm{deviatio}n_{\mathrm{air}}}$$

### Assessment of parent vessel and patency of the parent vessel

Two readers (CW, FDB) performed an independent assessment of in-stent stenosis. Segments scored as non-diagnostic due to impaired image quality on every reconstructed CT image were rated as potential sites for high-grade stenosis following the criteria of North American Symptomatic Carotid Endarterectomy Trial (NASCET).^12^

### DSA and DUS as the reference standard to assess in-stent vessel lumen

DSA was performed using standard techniques by interventional neuroradiologists. At least two projections of the in-stent vessel lumen were obtained, followed by an independent review by two readers (CW, FDB) blinded to the DSA reports.

DSA, DUS and PCD-CTA were performed in close temporal relationships (median of 1 day (0–4 d range)). Again, NASCET stenosis grades were derived from the angiographic view showing the greatest vessel narrowing. Board certified neurologists performed the standardised DUS. Only the highest measured peak systolic velocities were selected.

### Radiation dose assessment

All participants’ radiation dose parameters, specifically the dose length product and the dose area product were collected from the CT and DSA reports.

### Statistical analysis

IBM SPSS Statistics software (version 28.0) was used for statistical analysis. Normality of distributions for descriptive analysis was assessed using the Shapiro-Wilk test. Variables were expressed as mean ± standard deviation (SD) or median and interquartile ranges (IQR) as applicable. Likert scores were pooled across readers. Friedman test was used to compare different kernels for each reconstruction type (IOD, PE, VMI), with pairwise comparisons. For virtual monoenergetic imaging (VMI), the two keV levels were compared for each kernel before using the test. Friedman test was used to compare the Likert values of the different optimized reconstructions (IOD, PE, VMI).

Interobserver agreement was expressed in Cohen's kappa (κ) value and interpreted as follows: ≤ 0.20 as none, 0.21–0.40 as fair, and 0.41–0.60 as moderate, 0.61–0.80 as substantial, and ≥ 0.81 as almost perfect agreement.^13^ All p-values were corrected for multiple comparisons using the Bonferroni correction.

A 95% confidence interval range was calculated for all diagnostic accuracy tests, and a two-tailed p-value of < 0.05 was considered statistically significant.

Diagnostic performance of PCD-CTA compared with DSA and DUS imaging was evaluated using the pooled readers’ assessment of the vessel sites in patients. Receiver operating characteristic curve analysis was performed to calculate the area under the curve. Non-parametric distribution assumptions were made for standard error approximation.

Sensitivity, specificity, positive predictive value and negative predictive value were calculated for detecting parent vessel stenosis of ≥ 70% or residual stenosis.

## Results

Patient characteristics are detailed in Table 1. A total of 19 patients (64.3 ± 10.3 (mean patient age in years ± SD); 9 women) with carotid or subclavian artery stents and available DSA, DUS and PCD-CTA were analyzed.

**Table 1.** Patient characteristics.

Age (years)		64.3 ± 10.3
Sexe (male/female)		10 / 9
Stent location	Carotid artery	16
	Subclavian artery	3
Stent type	WallStent^®^	15
	CGuard^®^	2
	Omnilink^®^	3
Minimal in-stent luminal diameter in mm		3.5 ± 1.3
Distal luminal diameter in mm		5.1 ± 0.9
DAP in µGym^2^		1774.4 ± 3452 (1.5 mSv)
DLP in mGycm		1650 ± 71.0 (0.51 mSv)

*Note*: Except where indicated, data are numbers of participants, with percentages in parentheses.

Abbreviations: DAP: Dose area product, DLP: Dose length product, mGycm: Milligray centimeter, mGycm^2^: Milligray square centimeter, mm: millimeter; * Data are means ± standard deviations. The effective dose was calculated according to the recommendations of the IAEA (international atomic energy agency).

### Objective evaluation of image quality

VMI reconstructions showed a decrease in SNR and CNR for the two kernels and keV levels and with iMAR (for Bv56).

1. Reconstructions with 40 keV achieved the highest SNRs and CNRs, significantly outperforming the higher keV level (80 keV). For example, SNR for Bv56 at 40 keV was higher than at 80 keV in the parent vessel (mean ± SD: 21.7 ± 8.4 vs. 8.1 ± 4.3, p < 0.001), as well as in the in-stent vessel (median: 19.7 vs. 8.4, p < 0.001).
2. Reconstructions with smoother kernels (Bv56) resulted in higher SNRs and CNRs compared to harder kernels (Bv72) at 40 keV. As an example, SNR values for Bv56 and Bv72 kernels for 40 keV were in the parent vessel 21.7 (mean) vs. 11.3 (median, p < 0.001), as well as in the in-stent vessel 19.7 vs. 9.7 (mean, p = 0.03).
3. Reconstructions without iMAR showed comparable SNR and CNR in the parent vessel as well as in the in-stent vessel.

For IOD and PE reconstructions, smoother kernels consistently provided the highest SNRs in the parent vessel as well as in the in-stent vessel.

### Qualitative analysis

For VMI, images reconstructed with Bv56 at 40 keV achieved a higher score on the 5-point Likert scale than reconstructions with Bv72 (p < 0.001) in the proximal vessel as well as in the in-stent vessel.

**Figure 2.**
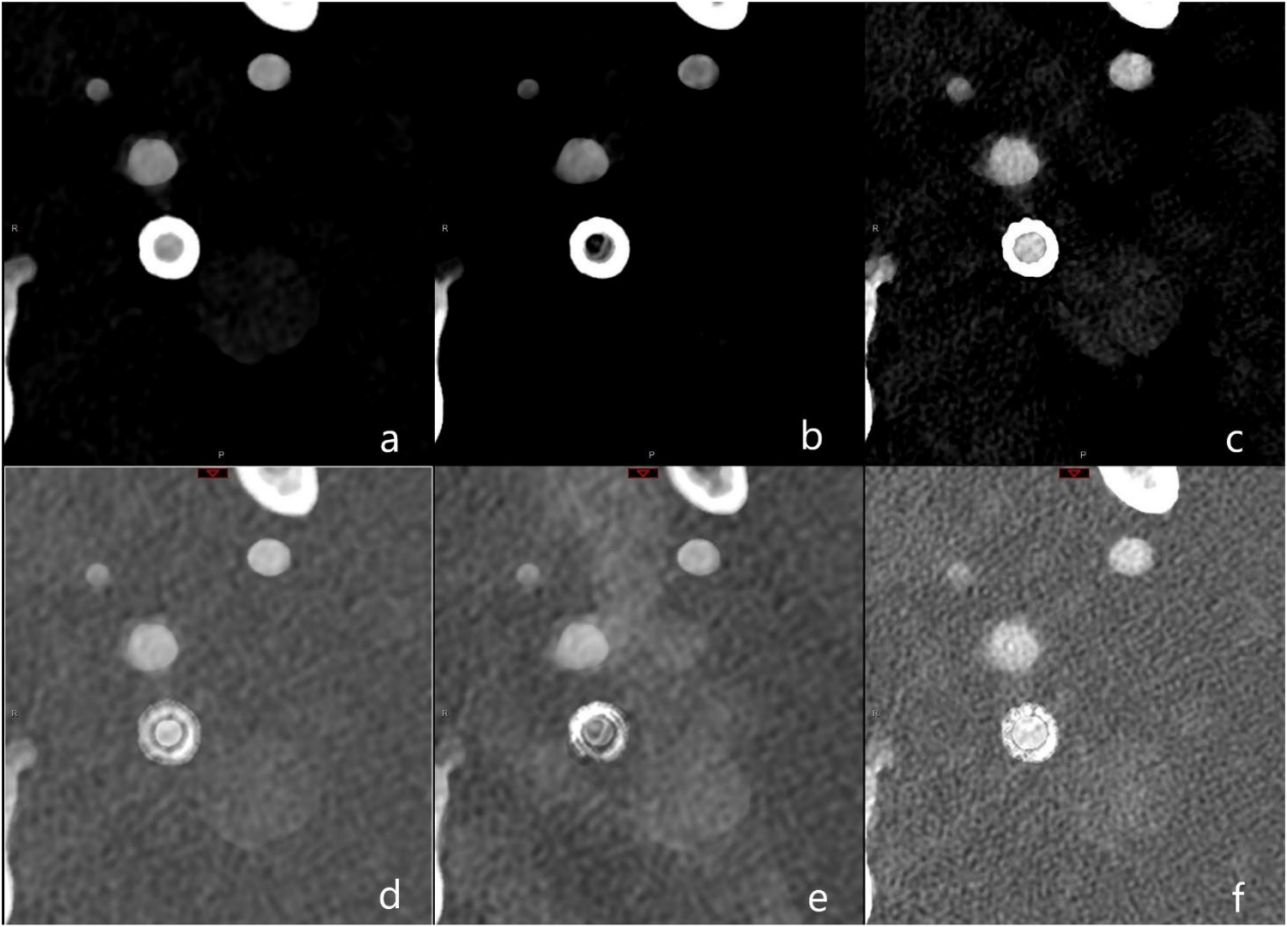
Illustration of reconstruction protocol for polyenergetic (PE) and iodine (IOD) reconstructions with two different kernels. In the first row PE and in the second row IOD are listed and in the three columns, from left to right Bv56 kernel without iMAR, Bv56 with iMAR and Bv72. In detail: a = Bv56, PE; b = Bv56 with iMAR, PE; c = Bv72, PE; d = Bv56, IOD; e = Bv56 with iMAR, IOD; f = Bv72, IOD. Abbreviations: Bv: Body vascular kernel, iMAR: iterative metal artifact reduction.

IMAR had no significant impact on image quality concerning evaluation of the proximal vessel or in-stent vessel lumen (p = 0.99) and even obscured the in-stent lumen as shown in Figure 2.

Similarly, PE and IOD reconstructed with Bv56 scored higher in the vessel proximal to the stent and in-stent vessel lumen compared to reconstructions with Bv72 independently of iMAR (Median score of iodine reconstructions at the in-stent vessel lumen with Bv56 and iMAR vs. Bv72: 4 vs. 3 respectively, p < 0.001).

Moderate agreement (κ = 0.71) was found between the two readers for qualitative ratings using the 5-point Likert-type scale. The internal consistency of the test, measured by Cronbach's alpha, was excellent (α = 0.82).

Detailed metrics of subjective and objective image quality are provided in the Supplemental Data.

When assessing the best spectral reconstructions to evaluate in-stent vessel lumen, pairwise comparisons identified virtual monoenergetic imaging reconstructions at 40 keV and iodine reconstructions as best to evaluate in-stent vessel lumen (p = 0.001).

### Diagnostic performance of PCD-CTA compared with DSA and DUS

In the pooled assessment of DSA of in-stent vessel lumen, 2 (11%) of the nineteen patients presented with relevant narrowing of the in-stent vessel lumen.

Compared to DSA imaging, the overall sensitivity of PCD-CT, considering all reconstructions for detecting 70%-stenosis, was 100% (2 of 2).

Inter observer agreement was substantial for image quality (*κ* *=* *0.79, p* *<* *0.001*) and excellent for diagnosis of in-stent stenosis (*κ* *=* *0.92, p* *<* *0.001*).

In 2/19 cases with elevated flow velocities in the in-stent vessel lumen, the subsequently performed PCD-CTA and DSA could rule out a flow relevant stenosis.

Compared to DUS as standard of care, the overall sensitivity of PCD-CT, considering all reconstructions for detecting 70%-stenosis, was 100% (2 of 2).

### Assessment of radiation dose

The mean dose-length product was 1650 ± 71.0 mGycm, and the dose-area product was 1774.4 ± 3452 µGym^2^, which correspond approximately to 0.51 mSv and (1.5 mSv) respectively.

## Discussion

In this study, we assessed the imaging possibilities of PCD-CTA for evaluating in-stent vessel lumen after carotid or subclavian artery stenting. The PCD-CTA was compared to DSA and DUS. PCD-CTA image reconstructions using smoother kernels and lower keV levels demonstrated superior SNR and CNR outperforming those with harder kernels and higher keV levels. This result appears foreseeable due to the physical circumstances; however, it contrasts with results on coronary and femoral stents as well as intracranial stents, which have shown better assessability with sharper kernels.^8,9,14^

This discrepancy could be due to the different physical properties, particularly the different density. Cobalt-chromium has a lower material density than platinum.

WallStents^®^ are made of cobalt-chromium-iron-nickel-molybdenum enhanced with a radiopaque tantalum core and Omnilink elite^®^ are made of cobalt chromium whereas flow diverters wires and intracranial stents have a platinum core. While stents for peripheral arteries have a similar material composition, the results regarding femoral stents were in an experimental setting in the form of a perfusion model in cadavers and only refer to polyenergetic reconstructions, which may limit the generalizability.^14^

Since coronary arteries and intracranial arteries have smaller vessel diameter than carotid arteries we considered the stent lumen width as a possible cause for heterogeneity. However, the subgroup analysis showed no differences in patients with minimal stent diameters of less than 2.5 mm residual diameter.

**Figure 3.**
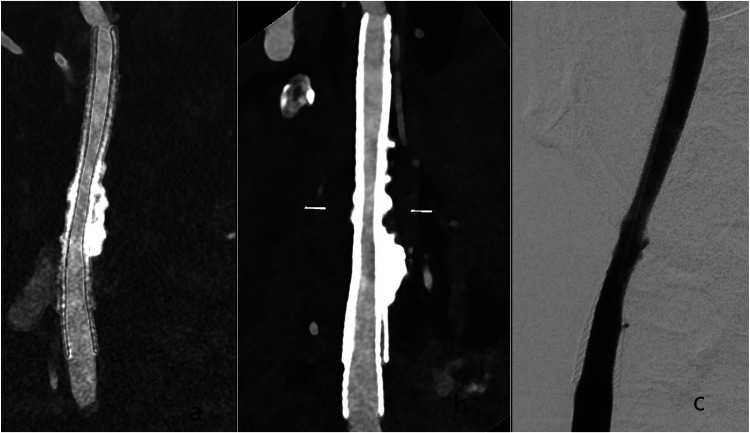
Postinterventional PCD-CTA with VMI and iodine reconstructions of an in-stent stenosis treated with a second carotid stent in a patient with calcified plaques at the carotid bifurcation. The patient has two carotid WallStents^®^ (double layer) in the right carotid artery. A is an iodine reconstruction with Bv56; B is an VMI with Bv56 at 40 keV with Bv56 of the right carotid artery; C is a p.a. 2D projections of the right carotid artery. Abbreviations: Bv: Body vascular kernel, keV: kiloelectron-volt, PCD-CTA: Photon-counting detector CT-Angiography, VMI: Virtual Monoenergetic Imaging.

Our results reproduce the work of other research groups on high negative predictive value of DUS in assessing in stent vessel lumen. Ultrasound is widely available, but PCD-CTA was able to resolve two cases of flow- relevant in-stent stenosis suspected in DUS in agreement with DSA. Dedicated reconstructions with the stated parameters of PCD-CTA yielded comparable results to DSA, as visualized in Figure 3.

In our study group the effective dose of PCD-CTA corresponded to a third of the DSA dose. As shown before in a different study setting, VMI reconstructions with low keV-levels may reduce iodine volume requirements by half.^15,16^

PCD-CTA could therefore be a possible low-radiation and non-invasive modality for clarifying abnormal flow measurements during duplex examinations. Instead of a DSA, an immediate clarification using PCD-CTA could be performed in the case of abnormal duplex sonography results (especially in non-symptomatic patients). This would not only be technically feasible with fewer personnel and less material but would also generally not require at least temporary patient monitoring. However, the continued high acquisition costs for a PCD-CT must be weighed against this.

### Limitations

Our results lack generalizability concerning the following points:

The study group comprised only a limited number of stents as well as a limited number of patients with in-stent-stenosis.

We observed some differences in visualization between different stent models, but the small number precludes any comparative conclusions.

Future studies on the diagnostic accuracy of photon-counting CT should include spectral reconstructions and a larger patient cohort, ideally with a larger variety of stents in larger multi-center cohorts and a prospective setting possibly with automated reconstructions and lumen quantification tools.

Additional to the higher spatial resolution, we consider spectral reconstructions in daily routine as a possibility for advancing image quality.

## Conclusion

PCD-CTA with spectral reconstructions and Bv56 kernels on a low keV level were best to evaluate the in-stent vessel lumen and demonstrated a high diagnostic accuracy for assessing in-stent vessel lumen in patients after carotid artery or subclavian artery stenting.

## Supplemental Material

sj-docx-1-ine-10.1177_15910199251374274 - Supplemental material for Photon-counting detector CT angiography to evaluate carotid and subclavian artery stents and compared to ultrasound and angiography – an in-vivo study with spectral reconstructions Supplemental material, sj-docx-1-ine-10.1177_15910199251374274 for Photon-counting detector CT angiography to evaluate carotid and subclavian artery stents and compared to ultrasound and angiography – an in-vivo study with spectral reconstructions by Frederic De Beukelaer, Laura L Wuyts, Steven van Hedent, Omid Nikoubashman, Iliana Kantzeli, Martin Wiesmann, Arno Reich, João Pinho, Hani Ridwan and Charlotte S Weyland in Interventional Neuroradiology

sj-docx-2-ine-10.1177_15910199251374274 - Supplemental material for Photon-counting detector CT angiography to evaluate carotid and subclavian artery stents and compared to ultrasound and angiography – an in-vivo study with spectral reconstructions Supplemental material, sj-docx-2-ine-10.1177_15910199251374274 for Photon-counting detector CT angiography to evaluate carotid and subclavian artery stents and compared to ultrasound and angiography – an in-vivo study with spectral reconstructions by Frederic De Beukelaer, Laura L Wuyts, Steven van Hedent, Omid Nikoubashman, Iliana Kantzeli, Martin Wiesmann, Arno Reich, João Pinho, Hani Ridwan and Charlotte S Weyland in Interventional Neuroradiology

## Abbreviations

Bv: Body vascular convolution kernel
CNR: Contrast-to-noise ratio
DSA: Digital Subtraction Angiography
DUS: Duplex Ultrasound
IOD: Iodine reconstruction
keV: Kiloelectron-volt
PCD-CTA: Photon-counting detector CT-Angiography
PE: Polyenergetic reconstruction
SNR: Signal-to-noise ratio
VMI: virtual monoenergetic imaging reconstruction
