# Supplementary material for: Photon-counting detector CT angiography to evaluate carotid and subclavian artery stents and compared to ultrasound and angiography – an in-vivo study with spectral reconstructions
Source: Interv Neuroradiol. 2025 Sep 8:15910199251374274. Online ahead of print. doi: 10.1177/15910199251374274 (PMC12417464; doi:10.1177/15910199251374274)
Supplement: sj-docx-1-ine-10.1177_15910199251374274 - Supplemental material for Photon-counting detector CT angiography to evaluate carotid and subclavian artery stents and compared to ultrasound and angiography – an in-vivo study with spectral reconstructions [file sj-docx-1-ine-10.1177_15910199251374274.docx]

Except for the tables of pairwise comparisons (which state p-values) all values are reported are means, medians are marked with a * (Bv: body vascular, CNR: Contrast-to-noise Ratio, IQR: Interquartile range, SD: standard deviation, SNR: signal- to-noise ratio)

**Quantitative image quality measurements of the PCD-CT.**

Table S1 Virtual monoenergetic reconstructions for Bv56 and Bv 72 kernel.

|  | Bv56 | |  | Bv56 and IMAR | |  | Bv72 | | p |
| --- | --- | --- | --- | --- | --- | --- | --- | --- | --- |
|  | keV 40 | keV 80 |  | keV 40 | keV 80 |  | keV 40 | keV 80 |  |
| Lumen of proximal artery | | | | | | | | | |
| SNR | 21,7* | 8,1* |  | 23,9 | 11,1* |  | 11,3* | 4,9* | <0.001 |
| CNR | 33,3 | 12,9 |  | 32,5 | 13,5 |  | 19,7 | 7,6 | <0.001 |
| Lumen of stent | | | | | | | | | |
| SNR | 19,7* | 8,4* |  | 14,2* | 6,7* |  | 9,7* | 3,5* | <0.001 |
| CNR | 36,0 | 15,1 |  | 31,3 | 17,1 |  | 21,5 | 9,6 | <0.001 |

Pairwise Comparisons VMI 40

| SNR | Bv72-Bv56 | Bv72-Bv56 IMAR | Bv56-Bv 56iMAR |
| --- | --- | --- | --- |
| Proximal vessel | <0.001 | <0.001 | 1 |
| In stent vessel | 0.012 | <0.001 | 0,4 |

Pairwise Comparisons VMI 40

| CNR | Bv72-Bv56 | Bv72-Bv56 IMAR | Bv56-Bv 56iMAR |
| --- | --- | --- | --- |
| Proximal vessel | <0.001 | <0.001 | 1 |
| In stent vessel | <0.001 | <0.001 | 1 |

Pairwise Comparisons VMI 80

| SNR | Bv72-Bv56 | Bv72-Bv56 IMAR | Bv56-Bv 56iMAR |
| --- | --- | --- | --- |
| Proximal vessel | <0.001 | <0.001 | 1 |
| In stent vessel | <0.001 | <0.001 | 0,45 |

Pairwise Comparisons VMI 80

| CNR | Bv72-Bv56 | Bv72-Bv56 IMAR | Bv56-Bv 56iMAR |
| --- | --- | --- | --- |
| Proximal vessel | <0.001 | <0.001 | 1 |
| In stent vessel | <0.001 | <0.001 | 1 |

Table S2. Iodine Reconstructions: Quantitative image quality measurements of the PCD-CT.

|  |  | Bv56 | Bv56 and IMAR | Bv72 | P |
| --- | --- | --- | --- | --- | --- |
| Lumen of proximal artery | | | | | |
| SNR |  | 14,2 | 13,7 | 8,5 | <0.001 |
| CNR |  | 156,8* | 289* | 294,9* | 0.03 |
| Lumen of stent | | | | | |
| SNR |  | 12,5* | 9,7* | 7,4 | <0.001 |
| CNR |  | 157,7* | 234,2 | 306,5* | 0,72 |

Pairwise Comparisons SNR

|  | Bv72-Bv56 | Bv72-Bv56 IMAR | Bv56-Bv 56iMAR | p |
| --- | --- | --- | --- | --- |
| Proximal vessel | <0.001 | <0.001 | 1 |  |
| In stent vessel | 0,34 | <0.001 | 0,04 |  |

Pairwise Comparisons CNR

|  | Bv72-Bv56 | Bv72-Bv56 IMAR | Bv56-Bv 56iMAR | p |
| --- | --- | --- | --- | --- |
| Proximal vessel | 0,25 | 0,03 | 1 |  |
|  |  |  |  |  |

Table S3. Polyenergetic Reconstructions: Quantitative image quality measurements of the PCD-CT.

|  | Bv56 | Bv56 and IMAR | Bv72 | P |
| --- | --- | --- | --- | --- |
| Lumen of proximal artery | | | | |
| SNR | 10,4* | 10,4* | 5,7* | <0.001 |
| CNR | 19,2* | 16,1 | 10,7 | <0.001 |
| Lumen of stent | | | | |
| SNR | 8,3* | 8,9 | 4,2* | <0.001 |
| CNR | 20,4 | 24,6 | 10,7 | <0.001 |

All values are reported are means, medians are marked with a * (Bv: body vascular, CNR: Contrast-to-noise Ratio, IQR: Interquartile range, SD: standard deviation, SNR: signal- to-noise ratio)

Pairwise Comparisons SNR

|  | Bv72-Bv56 | Bv72-Bv56 IMAR | Bv56-Bv 56iMAR |
| --- | --- | --- | --- |
| Proximal vessel | <0.001 | <0.001 | 0,18 |
| In stent vessel | <0.001 | <0.001 | 0.58 |

Pairwise Comparisons CNR

|  | Bv72-Bv56 | Bv72-Bv56 IMAR | Bv56-Bv 56iMAR |
| --- | --- | --- | --- |
| Proximal vessel | <0.001 | <0.001 | 0,18 |
| In stent vessel | <0.001 | <0.001 | 1 |

Comparisons of ROI

| SNR | PE56 | I56 | VMI56 40 | VMI56 80 | p |
| --- | --- | --- | --- | --- | --- |
| Proximal vessel | 10,4 | 14,2 | 21,7 | 8,1 | <0.001 |
| In stent vessel | 8,3 | 12,5 | 19,7 | 8,4 | <0.001 |

Pairwise comparison of reconstructions:

| SNR | I56-VMI5680 | I56-PE56 | I56-VMI5640 | VMI5680-PE56 | VMI5680-VMI5640 | PE56-VMI5640 |
| --- | --- | --- | --- | --- | --- | --- |
| Proximal vessel | 0.061 | 1 | <0.001 | 0.71 | <0.001 | <0.001 |
| In stent vessel | 0,003 | 0,344 | 0,002 | 0,71 | <0.001 | <0.001 |

| CNR | PE56 | I56 | VMI56 40 | VMI56 80 | p |
| --- | --- | --- | --- | --- | --- |
| Proximal vessel |  | 156,8 | 33,3 | 12,9 | <0.001 |
| In stent vessel |  |  | 36,0 | 15,1 | <0.001 |

Pairwise comparison of reconstructions:

| CNR | I56-VMI5680 | I56-PE56 | I56-VMI5640 | VMI5680-PE56 | VMI5680-VMI5640 | PE56-VMI5640 |
| --- | --- | --- | --- | --- | --- | --- |
| Proximal vessel | <0.001 | <0.001 | 0,044 | 0,083 | <0.001 | <0.001 |
| In stent vessel | <0.001 | <0.001 | 0,022 | 1 | <0.001 | 0.01 |

Except for the tables of pairwise comparisons all values are medians (Bv: body vascular, CNR: Contrast-to-noise Ratio, IQR: Interquartile range, SD: standard deviation, SNR: signal- to-noise ratio)

**Qualitative Assessment:**

Polyenergetic Reconstructions

|  | Bv56 | Bv56iMAR | Bv72 | P |
| --- | --- | --- | --- | --- |
| Proximal vessel | 4 | 4 | 3 | <0.001 |
| In stent vessel | 4 | 3 | 2 | <0.001 |

Pairwise comparison of reconstructions:

|  | Bv72-Bv56IMAR | Bv72-Bv56 | Bv56IMAR-Bv56 |
| --- | --- | --- | --- |
| Proximal vessel | <0.001 | <0.001 | 1 |
| In stent vessel | 0,11 | 0.001 | 0,291 |

Iodine Reconstructions

|  | Bv56 | Bv56iMAR | Bv72 | P |
| --- | --- | --- | --- | --- |
| Proximal vessel | 3 | 4 | 3 | <0.001 |
| In stent vessel | 3 | 2,5 | 2,5 | <0.001 |

Pairwise comparison of reconstructions:

|  | Bv72-Bv56IMAR | Bv72-Bv56 | Bv56IMAR-Bv56 |
| --- | --- | --- | --- |
| Proximal vessel | 0,007 | 0,028 | 1 |
| In stent vessel | 1 | 0,015 | 0,15 |

Virtual monoenergetic Reconstructions at 40keV

|  | Bv56 | Bv56iMAR | Bv72 | P |
| --- | --- | --- | --- | --- |
| Proximal vessel | 4 | 4 | 3.5 | <0.001 |
| In stent vessel | 4 | 4 | 4 | 0,101 |

Pairwise comparison of reconstructions:

|  | Bv72-Bv56IMAR | Bv72-Bv56 | Bv56IMAR-Bv56 |
| --- | --- | --- | --- |
| Proximal vessel | 0,012 | 0.001 | 1 |

Virtual monoenergetic Reconstructions at 80 keV

|  | Bv56 | Bv56iMAR | Bv72 | P |
| --- | --- | --- | --- | --- |
| Proximal vessel | 3 | 3 | 2,5 | <0.001 |
| In stent vessel | 3 | 2,5 | 2 | <0.001 |

Pairwise comparison of reconstructions:

|  | Bv72-Bv56IMAR | Bv72-Bv56 | Bv56IMAR-Bv56 |
| --- | --- | --- | --- |
| Proximal vessel | <0.001 | <0.001 | 1 |
| In stent vessel | 0,028 | 0.001 | 0,84 |

Comparisons of reconstructions

|  | PE56 | I56 | VMI56 40 | VMI7240 | p |
| --- | --- | --- | --- | --- | --- |
| Proximal vessel | 4 | 3 | 4 | 3,5 | 0,01 |
| In stent vessel | 4 | 3 | 4 | 4 | 0,01 |

Pairwise comparison of reconstructions:

|  | I56-VMI7240 | I56-PE56 | I56-VMI5640 | VMI7240-PE56 | VMI7240-VMI5640 | PE56-VMI5640 |
| --- | --- | --- | --- | --- | --- | --- |
| Proximal vessel | 1 | 0,037 | 0,001 | 0,302 | 0,003 | 0,705 |
| In stent vessel | 0,202 | 1 | 0,097 | 0,787 | 1 | 0,442 |
