# Supplementary material for: Photon-counting detector CT angiography to evaluate carotid and subclavian artery stents and compared to ultrasound and angiography – an in-vivo study with spectral reconstructions
Source: Interv Neuroradiol. 2025 Sep 8:15910199251374274. Online ahead of print. doi: 10.1177/15910199251374274 (PMC12417464; doi:10.1177/15910199251374274)
Supplement: sj-docx-2-ine-10.1177_15910199251374274 - Supplemental material for Photon-counting detector CT angiography to evaluate carotid and subclavian artery stents and compared to ultrasound and angiography – an in-vivo study with spectral reconstructions [file sj-docx-2-ine-10.1177_15910199251374274.docx]

**Scanning protocol**

Patients underwent a CTA centered on the implanted device acquired on a clinical first-generation PCD-CT scanner (NAEOTOM Alpha, Siemens Healthineers, Erlangen, Germany) operated in ultra-high-resolution (UHR) mode, resulting in a reconstructed slice thickness of 0.2 mm and slice increment of 0.1mm. Spectral reconstructions were reconstructed in 0.4 mm slice thickness and 0.2 mm slice increment. The reconstructed matrix size was 1024 x 1024, and the field of view was adjusted for each patient to optimally image the stented vessels.

The following acquisition parameters were used: tube voltage 140 or 120kvp, pitch 0.65, rotation time 0.5 s. CTA was performed after the administration of 80 ml iodinated contrast material (Ultravist-370 (generic name, iopromide; Bayer Healthcare, Berlin, Germany), injected through a 20-gauge intravenous antecubital vein catheter using a power injector. The flow rate 4ml/s. Opacification of the common carotid artery was monitored using a bolus tracking technique. The start time of data acquisition was determined with a fixed delay of five seconds after the attenuation threshold was reached. Iterative reconstruction (denoted “QIR” by the manufacturer) level 3 was used for all PCD-CTA images. Polyenergetic reconstructions in UHR-mode as well spectral reconstructions were performed for Iodine, Virtual monoenergetic images (VMI). Five image sets were reconstructed using the following vascular kernels: Bv56, Bv72. For Virtual monoenergetic Images-Reconstructions three keV levels (40, 80) were reconstructed for each kernel.
